# The role of the DNA damage response in zebrafish and cellular models of Diamond Blackfan anemia

**DOI:** 10.1242/dmm.015495

**Published:** 2014-05-08

**Authors:** Nadia Danilova, Elena Bibikova, Todd M. Covey, David Nathanson, Elizabeth Dimitrova, Yoan Konto, Anne Lindgren, Bertil Glader, Caius G. Radu, Kathleen M. Sakamoto, Shuo Lin

**Affiliations:** 1 Department of Molecular, Cell & Developmental Biology, University of California, Los Angeles, CA 90095, USA.; 2 Department of Pediatrics, Stanford University School of Medicine, Stanford, CA 94305-5208, USA.; 3 Department of Molecular and Medical Pharmacology, University of California, Los Angeles, CA 90095, USA.

**Keywords:** Ribosomal protein deficiency, Rps19, Rpl11, p53, ATR, RNR, Chk1, ATP, AMPK, Exogenous nucleosides

## Abstract

Ribosomal biogenesis involves the processing of pre-ribosomal RNA. A deficiency of some ribosomal proteins (RPs) impairs processing and causes Diamond Blackfan anemia (DBA), which is associated with anemia, congenital malformations and cancer. p53 mediates many features of DBA, but the mechanism of p53 activation remains unclear. Another hallmark of DBA is the upregulation of adenosine deaminase (ADA), indicating changes in nucleotide metabolism. In RP-deficient zebrafish, we found activation of both nucleotide catabolism and biosynthesis, which is consistent with the need to break and replace the faulty ribosomal RNA. We also found upregulation of deoxynucleotide triphosphate (dNTP) synthesis – a typical response to replication stress and DNA damage. Both RP-deficient zebrafish and human hematopoietic cells showed activation of the ATR/ATM-CHK1/CHK2/p53 pathway. Other features of RP deficiency included an imbalanced dNTP pool, ATP depletion and AMPK activation. Replication stress and DNA damage in cultured cells in non-DBA models can be decreased by exogenous nucleosides. Therefore, we treated RP-deficient zebrafish embryos with exogenous nucleosides and observed decreased activation of p53 and AMPK, reduced apoptosis, and rescue of hematopoiesis. Our data suggest that the DNA damage response contributes to p53 activation in cellular and zebrafish models of DBA. Furthermore, the rescue of RP-deficient zebrafish with exogenous nucleosides suggests that nucleoside supplements could be beneficial in the treatment of DBA.

## INTRODUCTION

Ribosome biogenesis is the most energy-consuming process in the cell (Thomas, 2000). It starts with the transcription of pre-ribosomal RNA (pre-rRNA) by RNA polymerase I (Kressler et al., 2010). The nascent pre-rRNA is assembled co-transcriptionally (Osheim et al., 2004) with a subset of ribosomal proteins (RPs) and other factors that facilitate pre-rRNA modification, cleavage and processing to 40S and 60S ribosomal subunits (Tafforeau et al., 2013). A growing set of genetic diseases is linked to mutations in genes that are involved in ribosome biogenesis (Narla and Ebert, 2010).

Mutations in some RPs impair the processing of pre-rRNA, leading to the accumulation of defective rRNAs in cells (Choesmel et al., 2007; Flygare et al., 2007; Léger-Silvestre et al., 2005). It causes the Minute phenotype in Drosophila and Diamond-Blackfan anemia (DBA) syndrome in humans (Lipton and Ellis, 2009; Marygold et al., 2007). RPS19 is the most frequently mutated RP in DBA (Boria et al., 2010; Draptchinskaia et al., 1999; Farrar et al., 2011). DBA is associated with anemia, malformations and predisposition to cancer. Physical abnormalities are especially frequent in individuals that have mutations in RPL11 and RPl5 (Gazda et al., 2008). Upregulation of the transcription factor p53 is involved in DBA pathogenesis as p53 inhibition ameliorates hematopoietic and developmental defects in animal models of DBA (Danilova et al., 2011; Danilova et al., 2008; Dutt et al., 2011; McGowan et al., 2008). Several mechanisms of p53 activation that suggest a special checkpoint controlling ribosomal biogenesis have been proposed, but the exact mechanism remains unclear (Fumagalli and Thomas, 2011).

A common diagnostic feature of DBA is upregulation of adenosine deaminase (ADA) (Fargo et al., 2013), which is also observed in animal models (Danilova et al., 2011; Danilova et al., 2008). ADA catabolizes ATP and dATP by removing an amino group from the adenine moiety. ADA deficiency leads to the accumulation of toxic deoxyadenosine and severe combined immunodeficiency syndrome (Sauer and Aiuti, 2009), whereas overexpression of ADA causes ATP depletion and hemolytic anemia (Chen and Mitchell, 1994). ADA overactivation in DBA suggests changes in nucleotide metabolism that might contribute to DBA pathophysiology. Although hemolysis is not a distinctive feature of DBA, the changes in plasma proteins indicate mild hemolytic anemia (Young and Alter, 1994). Other aspects of nucleotide metabolism might be changed in DBA, such as the balance of deoxynucleotide triphosphates (dNTPs), which has been shown in other models to be important for normal cell function (Bester et al., 2011; Hastak et al., 2008; Mannava et al., 2013). Nucleotide metabolism in DBA, however, has never been investigated.

**Fig. 1.**
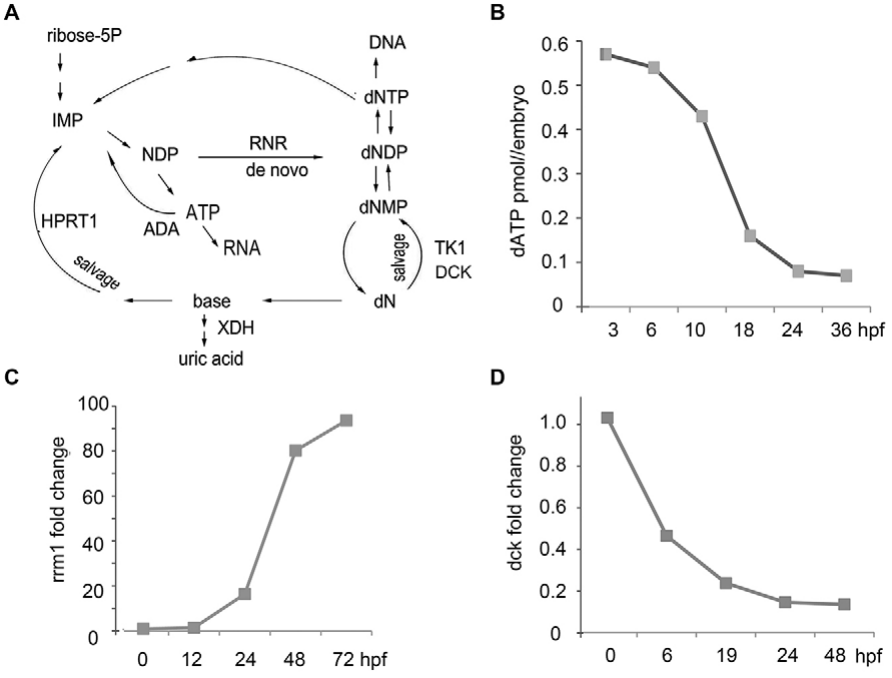
**Zebrafish embryos switch from a salvage to a *de novo* pathway of dNTP synthesis during development.** (A) dNTPs are synthesized *de novo* by the RNR enzyme or via the nucleoside salvage pathways. ADA, adenosine deaminase; NDP, nucleoside diphosphate; dNMP, dNDP and dNTP are deoxynucleoside mono-, di- and triphosphate, respectively; XDH, xanthine dehydrogenase; HPRT1, hypoxanthine phosphoribosyltransferase; TK1, thymidine kinase; DCK, deoxycytidine kinase. (B) Embryos have maternal supplies of dNTPs to support rapid cell division. The amount of free dATP in embryos decreased with age. hpf, hours post-fertilization. Means of three replicates were used to generate the graph. (C) *De novo* synthesis was low in early embryos; *rrm1* expression increased through development, as measured using RT-qPCR. The fold change of expression was calculated relative to the expression at 0 hpf. qPCR in panels C and D was performed in triplicate, and the means were used to generate the graphs. (D) The expression of *dck*, encoding the salvage enzyme, was high in early-stage embryos and decreased with age. The results of RT-qPCR analyses are shown. The fold change of expression was calculated relative to the expression at 0 hpf.

dNTPs are synthesized during the S phase of the cell cycle by ribonucleotide reductase (RNR), or through the nucleoside salvage pathways (Nordlund and Reichard, 2006) (Fig. 1A). Both dNTP deficiency and excess are dangerous for cells. Deficiency of any dNTP leads to replication stress and the activation of the ATR-p53 pathway, whereas an excess of dNTPs inhibits RNR and arrests proliferation (Kunz et al., 1994).

RNR is also induced in response to DNA damage in order to produce dNTPs for DNA repair (Chabes et al., 2003). RNR is a downstream target of ATR, the major kinase responsible for the DNA replication checkpoint (Branzei and Foiani, 2009; Elledge et al., 1993; Håkansson et al., 2006; Nordlund and Reichard, 2006). Salvage pathways of dNTP synthesis are also induced in response to DNA damage through the ATM kinase (Smal et al., 2006).

**TRANSLATIONAL IMPACT**

**Clinical issue**

Diamond Blackfan anemia (DBA) is a congenital disorder characterized by anemia, various malformations and an increased incidence of cancer. DBA is caused by mutations in ribosomal proteins (RPs), most frequently in RPS19, which interfere with rRNA processing and ribosome biogenesis. DBA is the most studied representative of several disorders that are associated with defects in ribosome biogenesis. Corticosteroids remain the major treatment option in DBA; hematopoietic stem cell transplantation is used in corticosteroid-resistant individuals. p53 activation appears to be a crucial mediator of many clinical features of DBA, but the molecular basis for p53 activation is unclear. A better understanding of the molecular mechanism underlying DBA is necessary for the development of new treatments for DBA and related disorders.

**Results**

A frequent feature in DBA is upregulation of adenosine deaminase, which suggests that DBA involves changes in nucleotide metabolism. Therefore, in this study, which uses zebrafish deficient in Rps19 and Rpl11 as models of DBA, the authors first examine nucleotide metabolism and show that enzymes involved in both nucleotide catabolism and biosynthesis are upregulated. RP deficiency also results in the arrest of cell proliferation. In spite of this, the authors find that both the expression of enzymes involved in dNTP biosynthesis and the incorporation of exogenous nucleotides into their DNA is increased in RP-deficient zebrafish. These findings point to increased DNA repair. In accordance with this interpretation, the authors find that markers for DNA damage are upregulated in RP-deficient zebrafish and in human fetal liver cells that are deficient in RPS19. Moreover, the application of inhibitors of the kinases involved in the DNA damage checkpoint decreases p53 upregulation and apoptosis and improves hematopoiesis in RP-deficient zebrafish. Finally, exogenous nucleosides also rescue RP-deficient zebrafish by resulting in the downregulation of pro-apoptotic p53 targets and a decrease in the activation of AMP-activated protein kinase.

**Implications and future directions**

These findings reveal the involvement of DNA damage pathways in the upregulation of p53 and in other molecular changes that are observed in a zebrafish model of DBA and in a human cellular model. They suggest that drugs that help to decrease DNA damage or that help to increase DNA repair might be effective in the treatment of DBA. They also suggest that nucleoside supplements work *in vivo* to increase DNA repair and might therefore be beneficial to individuals with DBA and related conditions.

Nucleotides are not only the building blocks for RNA and DNA but are also important signaling molecules; moreover, ATP is the major source of energy in cells. ATP shortage leads to activation of the energy sensor AMP-activated protein kinase (AMPK) (Hardie et al., 2012). Activated AMPK then switches on catabolic pathways to generate AT P and switches off biosynthetic pathways and cell-cycle progression.

In this study, we identify a link between RP deficiency, nucleotide metabolism and p53 activation. Our data suggest that zebrafish and human RP-deficient cells activate the ATR, ATM, CHK1 and CHK2, p53 pathway. We also uncover complex but coherent changes in nucleotide metabolism in RP-deficient cells that stems from their need to catabolize defective rRNAs, produce NTPs for more rRNAs and make extra dNTPs for DNA repair in response to ATR and ATM activation. RP-deficient cells exhibited an imbalance of dNTPs, ATP depletion, and upregulation of AMPK and p53. Although cells in RP-deficient zebrafish had a lower proliferation rate than controls, they incorporated more deoxycytidine into their DNA, suggesting the activation of nucleoside salvage pathways and increased DNA repair. A mixture of exogenous nucleosides rescued hematopoietic and morphological defects in RP-deficient zebrafish.

Our data suggest that activation of ATR, ATM, CHK1 and CHK2 contributes to p53 upregulation in DBA. The finding that exogenous nucleosides rescue RP-deficient zebrafish suggests that nucleoside supplements could be beneficial in individuals with DBA.

## RESULTS

### Embryos switch from salvage to *de novo* dNTPs synthesis during development

To explore the role of the DNA damage response in DBA, we first investigated nucleotide metabolism in wild-type and RP-deficient zebrafish embryos (Fig. 1A). Zebrafish eggs have a supply of maternal dNTPs, the amount of which decreases during embryo development, reaching a steady-state level at about 24 hours post-fertilization (hpf) (Fig. 1B). At the same time, *de novo* synthesis of dNTPs gradually increases, as illustrated by the expression of the gene *rrm1*, which encodes an RNR subunit (Fig. 1C). By contrast, expression of the gene *dck*, which encodes a salvage enzyme, decreases with age (Fig. 1D). Therefore, early embryos mostly recycle their nucleotides through the salvage pathways, whereas the *de novo* dNTP production gradually increases over time.

### RP deficiency changes the expression of genes involved in nucleotide metabolism

Rps19-deficient zebrafish were generated using a morpholino, as previously described (Danilova et al., 2008; Torihara et al., 2011; Uechi et al., 2008). We also used mutant *rpl11^hi3820bTg^* (Amsterdam et al., 2004), which has been characterized previously as a model of DBA in our lab (Danilova et al., 2011). Embryos have a supply of maternal ribosomes, and RP-deficient zebrafish can develop normally throughout early developmental stages. The morpholino generates Rps19 deficiency, which is most pronounced from 18 to 48 hpf (Danilova et al., 2008). Rpl11 mutants were predominantly used at 48 hpf – when at least 50% of Rpl11 protein remains (Danilova et al., 2011), mimicking haploinsufficiency of RPL11 in DBA. Most experiments were performed using both Rps19- and Rpl11-deficient models to reveal features common for deficiency of RPs from both small and large ribosomal subunits. Rps19 deficiency was also generated in a p53-null background, by using (*tp53^zdf1/zdf1^*) zebrafish (Berghmans et al., 2005).

**Fig. 2.**
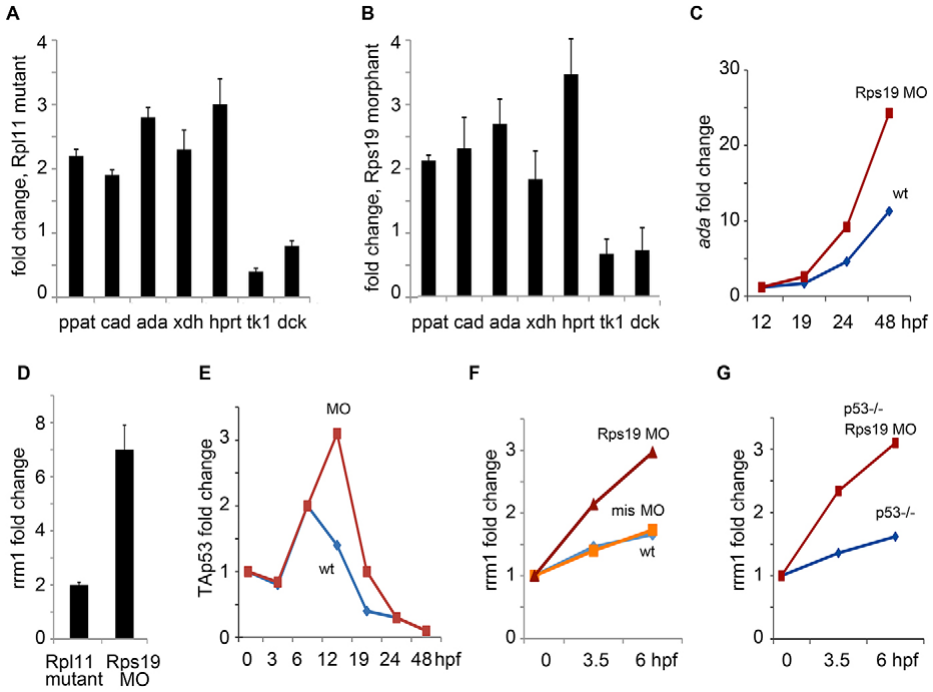
**The expression of genes involved in nucleotide metabolism changes in RP-deficient zebrafish.** (A) *ada* and *xdh*, which are involved in nucleotide catabolism, were upregulated in Rpl11 mutants. Genes encoding enzymes involved in nucleotide biosynthesis, such as *ppat* (phosphoribosyl pyrophosphate amidotransferase) and *cad* (carbamoyl-phosphate synthetase 2, aspartate transcarbamylase) were also upregulated. With regards to salvage enzymes, expression of *hprt* was increased. The levels of *tk1* and *dck*, which are expressed only in proliferating cells, were decreased. Gene expression was measured by using RT-qPCR analyses of embryos at 48 hpf. The fold change of expression in Rpl11 mutants was calculated relative to the expression in wild-type siblings. (B) Embryos that had been injected with a morpholino targeting Rps19 had similar changes in expression to those in Rpl11 mutants. RT-qPCR analyses were performed at 24 hpf. The fold change of gene expression in Rps19 morphants was calculated relative to expression of the gene wild-type embryos. (C) An increase in *ada* expression in Rps19-deficient embryos was detected, starting at 19 hpf. Red, embryos injected with a morpholino against Rps19 (Rps19 MO); blue, uninjected control (wt). Analyses were performed by using RT-qPCR. The fold change of expression was calculated relative to gene expression at 0 hpf. In panels C,E,F,G, qPCR was performed in triplicate and the means were used to generate the graphs. (D) *rrm1* was upregulated in Rpl11 mutants at 48 hpf and in Rps19 morphants at 24 hpf. Analyses were performed by using RT-qPCR. The fold change of gene expression for Rpl11 mutants was calculated relative to expression in wild-type siblings. The fold change in *ada* expression in Rps19 morphants was calculated relative to expression in wild-type embryos. In A,B,D, the means±s.d. are shown. (E) Timecourse of expression of a transactivating isoform of *tp53* (TAp53) in wild-type embryos (wt, blue) and in embryos that had been injected with Rps19 morpholino (MO, red). The results of RT-qPCR analyses are shown. The fold change of gene expression was calculated relative to expression at 0 hpf. (F) *rrm1* expression was increased at 3.5 and 6 hpf in embryos that had been injected with an Rps19-specific morpholino (red) but not in embryos that had been injected with a 5-base-mismatch morpholino (mis MO, orange). Expression of the gene in wild-type embryos is shown in blue. The results of RT-qPCR analyses are shown. The fold change of expression was calculated relative to expression of the gene at 0 hpf. (G) Upregulation of *rrm1* was also observed in *tp53*^−/−^ embryos that had been injected with an Rps19-specific morpholino (Rps19 MO, red), expression in control *tp53*^−/−^ embryos is in blue. The results of RT-qPCR analyses are shown. The fold change of expression was calculated relative to the expression at 0 hpf.

RP deficiency resulted in the upregulation of enzymes that are involved in both nucleotide catabolism and biosynthesis. In Rpl11 mutants, the expression of the genes encoding the catabolic enzymes Ada and Xdh was increased (Fig. 2A). *ada* and *xdh* were also upregulated in Rps19 morphants (Fig. 2B). These changes are consistent with the need to catabolize the aberrant rRNAs in RP-deficient cells and are in accord with the increased activity of ADA in DBA individuals (Fargo et al., 2013; Léger-Silvestre et al., 2005). Increased expression of *ada* in Rps19-deficient embryos was not mediated by p53, because *ada* was also increased in *tp53*−/− zebrafish (supplementary material Fig. S1). In wild-type embryos, *ada* expression increased during development, starting at 19 hpf, and the increase was higher in embryos that had been injected with a morpholino against Rps19 (Fig. 2C).

Both in Rpl11 mutants and Rps19 morphants, the expression of the *ppat* and *cad* genes, the products of which are involved in purine and pyrimidine biosynthesis, was increased (Fig. 2A,B), which is consistent with an increased demand for NTPs in RP-deficient cells that need to replace defective rRNAs. This notion is also supported by the increased expression of enzymes involved in nucleotide biosynthesis in DBA individuals (Halperin and Freedman, 1989) and by the increased expression of genes encoding factors involved in ribosome biogenesis in zebrafish Rpl11 mutants, these include PolI and PolII, Ddx family, and nucleolar factors among others (Danilova et al., 2011). Among salvage enzymes, the expression of *hprt1* was increased, whereas expression of *tk1* and *dck*, which are expressed only in growing cells, was decreased, consistent with the decreased proliferation of RP-deficient cells (Fig. 2A,B). Altogether, these results show that nucleotide metabolism is activated in RP-deficient zebrafish.

### Expression of RNR, which is involved in *de novo* dNTP synthesis, is upregulated in RP-deficient zebrafish

RP-deficient cells are characterized by proliferation arrest (Fumagalli et al., 2012). Therefore, it was an unexpected finding that the expression of the gene *rrm1*, encoding an RNR subunit, was increased 2- to 7-fold in RP-deficient zebrafish (Fig. 2D). RNR is a downstream target of ATR kinase, which is responsible for the replication stress checkpoint, thus indicating ATR activation. To study the possibility that p53 has an effect on *rrm1* upregulation, we compared timecourses of *tp53* and *rrm1* expression in Rps19-deficient zebrafish embryos. In wild-type embryos, expression of a transactivating isoform of *tp53* peaked at gastrulation (Fig. 2E). In Rps19-deficient embryos, this peak was much larger and remained high at later timepoints, but no difference in *tp53* expression was noted between wild-type and Rps19-morphant zebrafish until after 6 hpf. Conversely, the expression of *rrm1* had already increased in Rps19 morphants by 3.5 hpf and continued to increase at later timepoints (Fig. 2F). The injection of a 5-base-mismatch morpholino did not lead to *rrm1* upregulation (Fig. 2F). *rrm1* expression was also increased in *tp53*^−/−^ zebrafish that had been injected with the morpholino against Rps19 (Fig. 2G). Therefore, *rrm1* upregulation in Rps19-deficient zebrafish takes place soon after zebrafish embryos start zygotic transcription at ~3 hpf and is p53 independent.

### RP-deficient zebrafish upregulate the ATR/ATM-Chk1 pathway

**Fig. 3.**
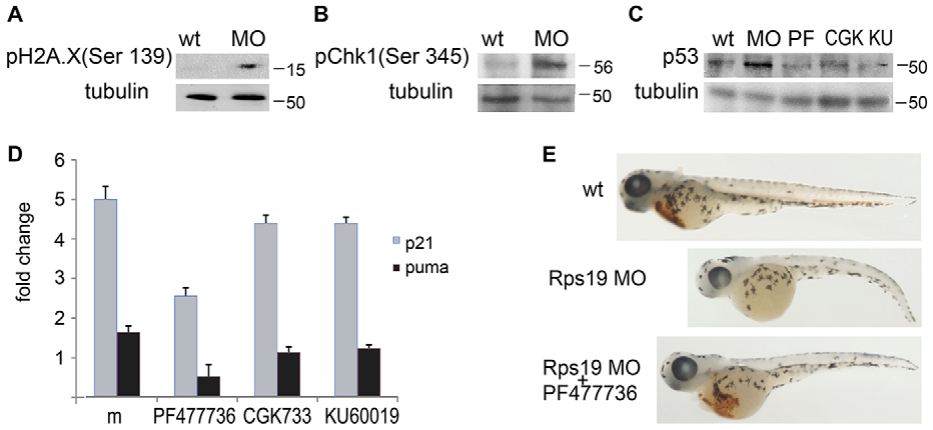
**RP-deficient zebrafish show activation of the DNA damage checkpoint pathway.** (A) Phosphorylation of residue Ser139 of histone H2A.X was induced in embryos that had been injected with an Rps19-specific morpholino (MO). Western blotting was performed at 24 hpf. Staining of tubulin was used for a loading control. Molecular masses are shown on the right. Wt, wild type. (B) Phosphorylation of residue Ser345 in Chk1 kinase was increased in Rps19-deficient embryos (MO). Western blotting was performed at 24 hpf. (C) Embryos that had been injected with an Rps19 morpholino had increased levels of p53; the treatment of morphants with 3 nM of Chk1 inhibitor PF477736 (PF), 10 nM of the ATR and ATM inhibitor CGK733 (GCK), or 3 nM of the ATM inhibitor KU60019 (KU) reduced p53 levels. Western blotting was performed at 24 hpf. (D) Treatment of Rpl11 mutants with inhibitors of the ATR-ATM-Chk1 pathway resulted in downregulation of the p53 targets *p21* and *puma*. Gene expression was analyzed by using RT-qPCR. The fold change of gene expression was calculated relative to expression in wild-type siblings. Means±s.d. are shown. (E) Embryos that had been injected with a morpholino against Rps19 (Rps19 MO) had few red blood cells at 3.5 days post-fertilization. The treatment of Rps19 morphants with PF477736 partially rescued this hematopoietic defect.

To determine whether *rrm1* upregulation in RP-deficient zebrafish embryos is caused by activation of the DNA damage checkpoints, we evaluated the phosphorylation of histone H2A.X at residue Ser139, which is a marker of DNA damage. Phosphorylation of H2A.X was not detectable in wild-type embryos but was induced in embryos that had been injected with Rps19-specific morpholino (Fig. 3A). Chk1 kinase acts downstream from ATR and ATM kinases to regulate the cell cycle in response to blocked DNA replication and genotoxic stress, and p53 is a Chk1 target. In RP-deficient zebrafish, we found increased phosphorylation of Chk1 at residue Ser345, a marker of Chk1 activation (Fig. 3B). To confirm Chk1 involvement in p53 activation, we treated Rps19-deficient embryos with a Chk1 inhibitor (PF477736) and found downregulation of p53 (Fig. 3C; supplementary material Fig. S2). The ATR and ATM inhibitor CGK733, and ATM inhibitor KU6009, also downregulated p53 in Rps19-deficient embryos. These inhibitors were also effective in Rpl11-mutant embryos, as shown by downregulation of the p53 targets *p21* and *puma* in Rpl11 mutants (Fig. 3D). Treatment with these inhibitors decreased the severity of morphological defects, improved survival (supplementary material Fig. S3) and increased the amount of red blood cells in RP-deficient embryos (Fig. 3E). These data pointed to activation of the ATR/ATM-Chk1/Chk2/p53 pathway in RP-deficient zebrafish.

### DNA damage checkpoints are activated in RPS19-deficient human fetal liver cells

**Fig. 4.**
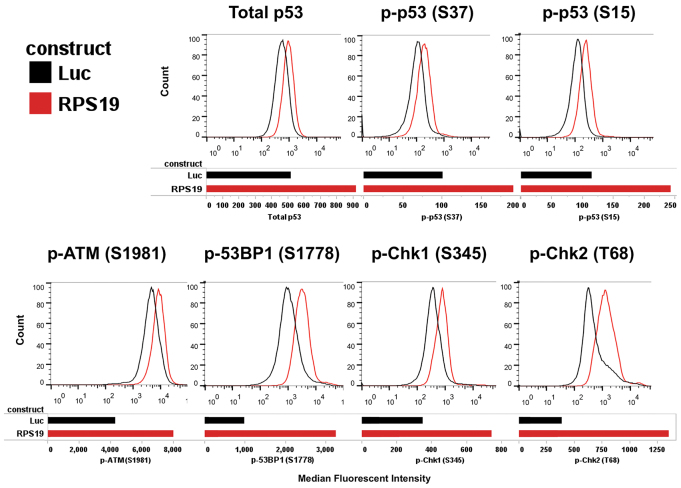
**Human RPS19 deficient cells have an activated DNA damage checkpoint.** Intracellular phosphoflow cytometry of RPS19-deficient human CD34^+^ cells from fetal liver 5 days after transduction with lentiviral vectors expressing shRNA against *RPS19*. A vector targeting luciferase was used as a control. All vectors also expressed GFP. Histograms show GFP-positive gated cells from luciferase control (Luc, black) or RPS19-knockdown cells (RPS19, red). Bar graphs show the median fluorescent intensity obtained from the histograms for each antibody-fluorochrome conjugate. We used primary antibodies against phosphorylated p53 at residue S37 [p-p53 (S37)], total p53, phosphorylated p53 at residue S15 [p-p53 (S15)] that were conjugated to Alexa Fluor 647. We also used the following unconjugated antibodies: mouse against phosphorylated ATM at residue S1981 [p-ATM (S1981)], rabbit against phosphorylated 53BP1 at residue S1778 [p-53BP1 (S1778)], rabbit against phosphorylated Chk1 at S345 [p-Chk1 (S345)], and rabbit against phosphorylated Chk2 at T68 [pChk2 (T68)] with a secondary labeling step using either anti-mouse IgG antibody conjugated to PE or anti-rabbit IgG antibody conjugated to PE.

Next, we examined whether RPS19-deficient human fetal liver cells also show signs of DNA damage response. Previously, a cellular model of DBA has been developed that utilized transduction of human CD34^+^ cells from umbilical cord blood and bone marrow with lentiviral vectors expressing short hairpin RNA (shRNA) against *RPS19* (Flygare et al., 2005). We used the same vectors to transduce human CD34^+^ fetal liver cells. Transduction with the shRNA vectors strongly reduced the *RPS19* mRNA levels, reduced levels of the protein and induced expression of p53 targets, such as *p21* (supplementary material Fig. S4). The level of the RPS19 protein decreased more slowly than that of the mRNA, so that 5 days after transduction the cells still had ~50% of RPS19 protein relative to controls, which is similar to previous reports (Flygare et al., 2005). At that stage, we determined the expression of markers of DNA damage checkpoint pathways using corresponding antibodies and the phosphoflow technique. As a positive control for DNA damage, we treated our cells with etoposide (supplementary material Fig. S5). We found a consistent response that involved an increase in p53 phosphorylation at residues Ser15 and Ser37, phosphorylation of ATM at residue Ser1981, phosphorylation of 53BP1 at residue Ser1778, phosphorylation of Chk1 at residue Ser345 and phosphorylation of Chk2 at residue Thr68 (Fig. 4). Upregulation of these markers was proportional to RPS19 downregulation (supplementary material Fig. S6). These data indicate that RP-deficient cells function under conditions of chronic DNA damage.

### Rps19- and Rpl11-deficient zebrafish have imbalanced dNTP pools

**Fig. 5.**
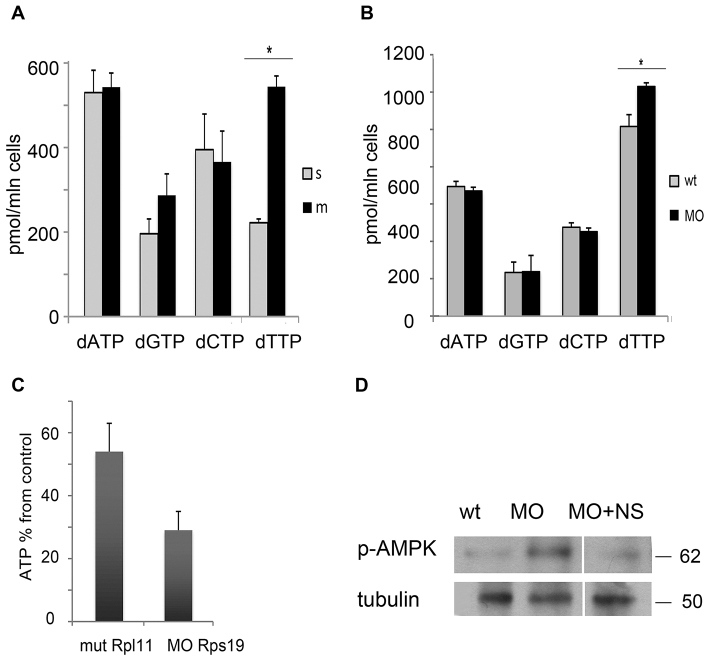
**RP-deficiency results in an imbalanced dNTP pool, ATP depletion and AMPK activation.** (A) Levels of dNTPs in Rpl11 mutants (m, black) and wild-type siblings (s, gray) at 48 hpf. dTTP was increased in Rpl11 mutants. Student’s *t*-test, *P<0.01. (B) At 18 hpf, dTTP was increased in embryos that had been injected with an Rps19 morpholino (MO, black). Student’s *t*-test, *P<0.05. wt (gray), wild-type embryos. (C) The ATP levels in Rpl11 mutants (mut Rpl11) and Rps19 morphants (MO Rps19) were decreased. The ATP level is shown relative to that of siblings for Rpl11 mutants, and relative to that of wild-type embryos for Rps19 morphants. ATP levels were measured 48 hpf in mutants and 24 hpf in morphants. In A–C, the means±s.d. are shown. (D) Rps19 deficiency led to the activating phosphorylation of AMPK at Thr172 (p-AMPK), as analyzed by western blotting. Treatment with nucleosides reduced AMPK phosphorylation. wt, wild-type embryos; MO, embryos that had been injected with a morpholino against Rps19. Molecular masses are indicated on the right.

In order to analyze the consequences of changes in the expression of enzymes involved in nucleotide metabolism and activation of the DNA damage response in RP-deficient zebrafish, we measured concentrations of dNTPs in Rpl11 mutants and Rps19 morphants. We found that the levels of dTTP were increased in RP-deficient zebrafish embryos, dATP and dGTP were not significantly altered, and dCTP levels were slightly decreased compared with those of controls (Fig. 5A,B). Therefore, Rps19- and Rpl11-deficient zebrafish have imbalanced dNTP pools, suggesting that RP-deficient cells are incapable of maintaining a proper balance of nucleotides.

### ATP levels are lower in RP-deficient zebrafish

It is known that upregulation of ADA results in ATP depletion (Chen and Mitchell, 1994) and ADA is upregulated in our DBA models. ATP is produced by glycolysis, which is suppressed in our DBA models due to p53 upregulation (Danilova et al., 2011). Therefore, both of these factors might affect ATP concentrations in RP-deficient zebrafish. We measured ATP levels in Rpl11 mutants and in zebrafish embryos that had been injected with a morpholino against Rps19, and we found decreased amounts of ATP in both models (Fig. 5C).

### AMPK is activated in RP-deficient zebrafish

AMPK senses cellular energy levels (Hardie et al., 2012). Low ATP levels signal metabolic stress to AMPK, leading to its phosphorylation at residue Thr172 and activation. We found that, in Rps19-deficient embryos, AMPK was phosphorylated at residue Thr172, indicating that it was activated (Fig. 5D). p53 is an AMPK target (Jones et al., 2005), suggesting that in RP-deficient cells, AMPK contributes to p53 activation. The activation of AMPK explains the decreased expression of genes that are involved in the biosynthesis of lipids and proteins in Rpl11-mutant zebrafish (Danilova et al., 2011).

### Nucleoside salvage pathways are activated in RP-deficient zebrafish

**Fig. 6.**
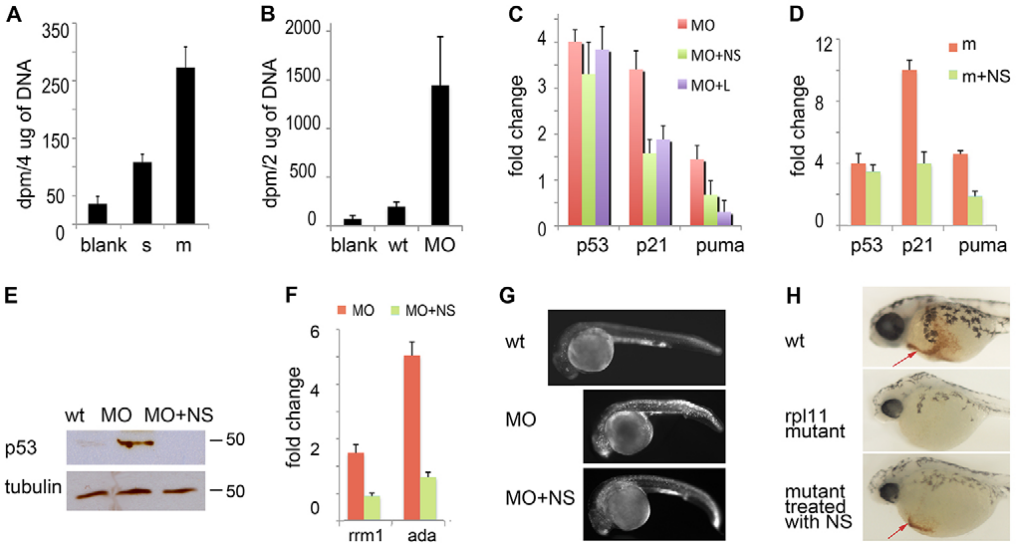
**Rescue of RP-deficient zebrafish with exogenous nucleosides.** (A) At 48 hpf, Rpl11 mutants incorporated more [^3^H] deoxycytidine into DNA than their wild-type siblings (*P*<0.002). The count adjusted to 4 μg of genomic DNA is shown. (B) At 48 hpf, embryos that had been injected with a morpholino against Rps19 incorporated more [^3^H]deoxycytidine into DNA than wild-type embryos (*P*<0.005). The count adjusted to 2 μg of genomic DNA is shown. (C) Supplementation with exogenous nucleosides (NS) decreased the expression of *tp53*, *p21* and *puma* in zebrafish embryos that had been injected with a morpholino against Rps19 (MO). L, treatment with 0.5 mg/ml leucine was used for comparison. RT-qPCR analyses were performed at 22 hpf. The fold change in gene expression was calculated relative to expression in wild-type embryos. (D) The addition of exogenous nucleosides reduced the expression of *tp53*, *p21* and *puma* in Rpl11-mutant zebrafish embryos. RT-qPCR analyses were performed at 48 hpf. The fold change in expression was calculated relative to expression in wild-type siblings. (E) Nucleoside treatment decreased the level of p53 protein in Rps19-deficient zebrafish embryos. 22 hpf. Western blot. (F) The addition of exogenous nucleosides normalized the altered expression of genes that are involved in nucleotide metabolism – *rrm1* and *ada*. RT-qPCR analyses were performed at 22 hpf. In A–D and F, means±s.d. are shown. (G) Treatment with nucleosides decreased the amount of apoptosis. Embryos were injected with a morpholino against Rps19, and half of these were treated with nucleosides. Representative images are shown of fish at 28 hpf. Acridine orange staining shows fewer apoptotic cells in morphants that had been treated with nucleosides. (H) The addition of exogenous nucleosides increased the amount of red blood cells in Rpl11 mutants. Representative images are shown of O-dianizidine staining of fish at 80 hpf. Rpl11 mutants were confirmed by genotyping after staining. Arrows point to erythroid cells.

DNA damage stimulates the production of dNTPs not only through the *de novo* pathway by upregulation of RNR, but also through activation of the salvage pathways. Specifically, ATM kinase activates DCK by phosphorylation on residue Ser74 in response to DNA damage (Smal et al., 2006; Yang et al., 2012), DCK then catalyzes phosphorylation of deoxynucleosides. In the absence of stress, only a small percentage of nucleotides in DNA originate from salvage pathways. In RP-deficient zebrafish, we observed increased incorporation of [^3^H]deoxycytidine into DNA. Rpl11-mutant fish incorporated 2.5-fold more deoxycytidine into DNA than their wild-type siblings (Fig. 6A), whereas embryos that had been injected with a morpholino against Rps19 incorporated sevenfold more deoxycytidine in comparison with controls (Fig. 6B). These findings point to activation of the Dck enzyme and, furthermore, suggest activation of its upstream kinase ATM in RP-deficient zebrafish. Because RP-deficient zebrafish activated nucleotide salvage pathways, this suggests that nucleosides could be used as a treatment to decrease DNA damage in these zebrafish.

### Exogenous nucleosides rescue RP-deficient zebrafish

Previously, exogenous supply of nucleosides has been shown to rescue cells experiencing oncogene-induced replication stress (Bester et al., 2011) and senescence (Mannava et al., 2013), and to downregulate p53 in the Caco-2 cell line (Ortega et al., 2011). Nucleosides, however, have never been used *in vivo* for this purpose. Therefore, we treated Rpl11-mutant and Rps19-morphant zebrafish with a mixture of deoxyadenosine, deoxyguanosine, deoxycytidine and thymidine at concentrations of 10, 25, 50 and 100 μM. The 50 μM concentration was chosen as being optimal for subsequent treatments. The efficiency of *rps19* downregulation by the morpholino was not affected by treatment with nucleosides (supplementary material Fig. S7). We also found that treatment with nucleosides downregulated the expression of p53 and p53 targets (Fig. 6C,D). For comparison, we used treatment with leucine, which has been shown to rescue animal and cellular DBA models (Jaako et al., 2012; Payne et al., 2012). *tp53* mRNA was only slightly decreased after nucleoside treatment, but at the protein level, the effect was more pronounced (Fig. 6E). Treatment with nucleosides also decreased the level of AMPK phosphorylation (Fig. 5D), indicating downregulation of its activity.

The expression of the genes encoding the enzymes involved in nucleotide metabolism, such as *rrm1* and *ada*, returned closer to normal levels after treatment with exogenous nucleosides (Fig. 6F).

Zebrafish that had been injected with a morpholino against Rps19 had high levels of apoptosis at 24–30 hpf, especially in the head and blood (Fig. 6G). Apoptosis is part of normal development, but it peaks at earlier stages, and only a few apoptotic cells are seen after 19 hpf in wild-type embryos. Treatment with nucleosides resulted in a reduction of the number of apoptotic cells (Fig. 6G; supplementary material Fig. S8). The treatment increased the amount of red blood cells in Rps19-deficient zebrafish (Fig. 6H); of 40 Rpl11-mutant embryos, 36 (90%) did not have blood cells at 80 hpf, and after the supply of exogenous nucleosides, blood was present in 29 (72%) embryos.

Treatment with nucleosides also improved survival and decreased morphological defects in RP-deficient zebrafish (supplementary material Fig. S9). These effects suggest that exogenous nucleosides provided sufficient dNTPs for DNA repair and reduced stress in RP-deficient zebrafish. A treatment comprising a mixture of adenosine, guanosine, uridine, cytidine and thymidine was slightly less efficient than treatment with deoxyribonucleosides (supplementary material Fig. S10), but also rescued RP-deficient zebrafish embryos, pointing to the activation of multiple nucleoside salvage pathways in these embryos.

## DISCUSSION

**Fig. 7.**
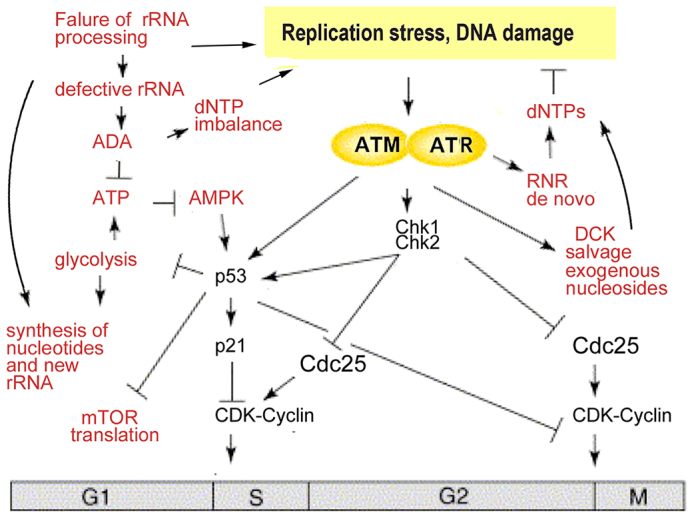
**Schematic of the changes that are induced in cells by a deficiency of RPs.** The nascent rRNA cannot be processed correctly in the absence of some RPs, which hypothetically leads to replication stress and DNA damage. Alongside this stress, global changes in nucleotide metabolism arise from (i) the necessity to catabolise defective rRNAs; (ii) the need to produce more rRNAs; (iii) the need to produce more dNTPs for DNA repair; (iv) the decreased availability of ATP and precursors for biosynthesis, caused by suppressed glycolysis; and (v) ADA activity destroying ATP. Altogether, metabolism perturbations lead to dNTP imbalance and ATP depletion. These factors can further exacerbate replication stress and DNA damage. The pattern of p53 phosphorylation that we observed is consistent with inputs from several kinases from the ATR-ATM-Chk1-Chk2 pathway. In addition, the activation of AMPK, caused by low ATP levels, can contribute to p53 activation. During replication stress and DNA damage, ATR kinase activates RNR (*de novo* pathway) to increase production of dNTPs, which are necessary for DNA repair. At the same time, ATM kinase activates the salvage enzyme DCK to produce more dNTPs through salvage pathways. Salvage pathways are barely used in healthy cells, but for stressed cells they are much more important, as illustrated by the increased incorporation of radioactively labeled deoxycytidine into the DNA of zebrafish that are deficient in RPs. Exogenous nucleosides rescue cells that are deficient in RPs by decreasing replication stress through providing additional dNTPs for DNA repair.

Our data suggest that failure of ribosome biogenesis in RP-deficient cells results in upregulation of the ATR-ATM-Chk1-Chk2-p53 pathway (Fig. 7). Upregulation of *rrm1* in the early stages of the development of RP-deficient zebrafish suggests that activation of ATR takes place soon after the start of zygotic transcription, before p53 upregulation. The exact mechanism of ATR and ATM upregulation in RP-deficient cells requires further studies, especially the role of aberrant processing of pre-rRNA. Several reports have shown that pre-rRNA processing starts before its transcription is finished (Huertas and Aguilera, 2003; Osheim et al., 2004; Schneider et al., 2007). The architectural organization of ribosomal (r)DNA is very complex, and obstructions of pre-rRNA processing have been suggested to interfere with its transcription and ultimately with replication (Bermejo et al., 2012). In addition, the increased demand for rRNA in RP-deficient cells might hyper-activate rDNA units, increasing the probability of topological stress occurring that would result in fork reversal and R-loop accumulation (Bermejo et al., 2012). Transcriptional defects might lead to transcription elongation impairment, increased formation of DNA-RNA hybrids, breaks and hyper-recombination, which will impair replication (Bermejo et al., 2012; Huertas and Aguilera, 2003). Similarly, mRNA splicing happens co-transcriptionally, and splicing defects increase the formation of DNA double-strand breaks (Li and Manley, 2005; Wahba et al., 2011).

The ATR-Chk1 axis has recently been implicated in cell-cycle arrest that is induced by a prolonged treatment with low levels of actinomycin D, a selective inhibitor of rRNA synthesis (Ma and Pederson, 2013). The exact mechanism of ATR induction by this treatment was not uncovered.

The phosphorylation of p53 at residues Ser15 and Ser37 observed in RPS19-deficient human CD34^+^ fetal liver cells is probably performed by multiple protein kinases, including ATR, ATM, Chk1 and Chk2, the activation of which was observed in these cells. Phosphorylation at these sites is known to stabilize and activate p53, but other mechanisms could also contribute to p53 activation (Ashcroft et al., 2000; Lane and Levine, 2010).

Changes in the expression of enzymes involved in nucleotide metabolism in models of DBA are consistent with the requirement of RP-deficient cells to catabolize the defective rRNA, produce more nucleotides to make new rRNAs and to make increased amounts of dNTPs sufficient for DNA repair. Activity of the salvage enzyme DCK is regulated at the post-translational level. DCK is activated by phosphorylation in response to DNA damage (Smal et al., 2006; Yang et al., 2012), and increased incorporation of radioactive deoxycytidine into the DNA of RP-deficient zebrafish suggests that Dck activity was increased in our models.

Decreased or imbalanced dNTP pools can be a source of DNA damage (Bester et al., 2011; Hastak et al., 2008; Kunz et al., 1994; Mannava et al., 2013). In RP-deficient zebrafish, we found changes in the dNTP pool composition, most notably, a significant increase in dTTP levels. Elevated dTTP can induce replication stress through the induction of a relative dCTP deficiency (Austin et al., 2012). Similarly, disproportional increases in dCTP and decreases in dTTP have been shown to result in replication fork collapse and genomic instability (Sánchez et al., 2012). Therefore, although an imbalance of the dNTP pool can be a result of the efforts of a cell to compensate for ribosomal stress and DNA damage, such an imbalance can induce further damage.

Upregulation of the ADA enzyme is one of the most important changes in RP-deficient cells because ADA can deplete the ATP pool and lead to AMPK activation. Indeed, we found decreased ATP levels and increased AMPK phosphorylation at residue Thr172 in Rps19-deficient zebrafish. Activated AMPK can phosphorylate p53 at residue Ser15, contributing to the activation of p53 by the ATR-ATM pathway.

One of the consequences of p53 activation is the suppression of glycolysis (Matoba et al., 2006), which we also observe in Rpl11-mutant fish (Danilova et al., 2011). When p53 expression is low, such as in tumor cells, glycolysis is increased to provide biosynthetic intermediates for fast growing tumor cells, this is known as the Warburg effect. Glycolysis is also increased in normal cells during proliferation (Lunt and Vander Heiden, 2011). One of the main cellular energy expenditures is the production of nucleotides for RNA and DNA. DNA synthesis is strongly dependent on glycolysis, thus the S phase of the cell cycle is accompanied by a surge of glycolysis (Lunt and Vander Heiden, 2011), and suppression of glycolysis results in decreased production of AT P and precursors for biosynthesis (Lunt and Vander Heiden, 2011). Decreased production of ATP due to suppressed glycolysis would synergize with overactivated ADA in decreasing the ATP pool in RP-deficient cells and activating AMPK.

Activated AMPK induces energy saving measures in the cell, which includes inhibition of translation. Pre-rRNA processing requires hundreds of various proteins, so when translation is inhibited, fewer such proteins are produced and supplied to ribosomes (Talkish et al., 2012). This might be one of the reasons why treatment with leucine improves anemia in animal models of DBA (Jaako et al., 2012; Payne et al., 2012). However, overactivation of mTOR, especially for prolonged periods of time, might lead to cellular transformation.

Here, exogenous nucleosides rescued RP-deficient zebrafish. In healthy cells, the dNTP pool is limited and, in response to DNA damage, cells activate RNR to produce more dNTPs. However, when cells fail to produce a balanced pool through *de novo* pathways, salvage pathways might become essential. We found that RP-deficient zebrafish incorporated 2.5- to 7-fold more exogenous deoxycytidine into their DNA than controls. Because proliferation is arrested in RP-deficient cells, the increase must be due to DNA repair. In support of the ability of salvage pathways to compensate for the deficiency of *de novo* nucleotide biosynthesis, exogenous deoxynucleosides have been shown to facilitate DNA repair during ribonucleotide reductase blockade in cancer cells (Kunos et al., 2011) and to reduce replication stress and DNA damage that is induced by oncogenes (Bester et al., 2011; Burrell et al., 2013; Mannava et al., 2013).

Previous studies of nucleotide biosynthesis suggest that only a small fraction of dietary nucleosides and nucleotides become incorporated into DNA. Our data, together with recent data from other labs, suggest that exogenous dietary sources might become vital in a state of genotoxic stress. Blood cells depend more on salvage pathways for the production of dNTPs than other cells (Austin et al., 2012); thus, they are more vulnerable to the adverse effects of a stressed metabolism. RP-deficient red blood cells have increased activation of p53 in comparison to other cell types (Dutt et al., 2011); therefore, they might benefit the most from exogenous nucleosides.

Our findings suggest that individuals with DBA can benefit from nucleoside supplements. Nucleoside mixtures are safe, and many infant formulas already include nucleosides and nucleotides (Singhal et al., 2010). Nucleoside supplementation in enteral nutrition has been shown to improve outcomes in physiologically stressed individuals (Hess and Greenberg, 2012).

Nucleoside supplementation might be beneficial not only in DBA but in many other conditions that involve activation of DNA damage checkpoints. For example, we found that treatment with nucleosides improved the survival of irradiated zebrafish (N.D. and S.L., unpublished data). Many genetic disorders are associated with the activation of DNA damage checkpoints; therefore, nucleoside supplementation might find wide therapeutic application.

## MATERIALS AND METHODS

### Zebrafish

Zebrafish *(Danio rerio)* lines used: AB, *rpl11^hi3820bTg^* and *tp53^zdf1/zdf1^*. Embryos were obtained by natural spawning. The University of California, Los Angeles Animal Committee approved the study.

### Genotyping zebrafish embryos with a mutation in *rpl11*

Individual zebrafish embryos were placed into 20 μl of 50 mM NaOH and heated for 20 minutes at 95^°^C to dissolve them. The lysates were neutralized with 2 μl of 1 M Tris-HCl buffer, pH 8 and 1 μl of each lysate was used in PCR analyses. Genotyping was performed with primers that had been used in the initial screen (Amsterdam et al., 2004); forward primer 5′-CTCTTCTAGTGATCAAACATGGCG-3′ from the first exon, and reverse primer 5′-GCTAGCTTGCCAAACCTACAGGT-3′ corresponding to the viral insertion in the first intron. The forward primer described above and a reverse primer from the first intron 5′-TGCTCATCCGGAATCTGTACA-3′ (corresponding to the unmodified genomic sequence) were used in PCR analyses to discriminate between homozygous and heterozygous embryos.

### Human fetal liver cells

Human fetal liver tissues were obtained from Advanced Bioscience Resources (Alameda, CA). Cells were sorted for CD34^+^ using MACS cell separation (Miltenyi Biotec, Auburn, CA) and then grown in x-Vivo15 medium (Lonza, Basel, Switzerland) containing 10% fetal bovine serum, 50 ng/ml of FLT-3 ligand, thrombopoietin and stem cell factor, and 20 ng/ml of IL-3 and IL-6. Cells were transduced with lentivirus containing shRNA against RPS19 or luciferase, sorted for green fluorescent protein (GFP) 72 hours later and then harvested at 5 days post-transduction.

### RT-qPCR

RNA for reverse-transcription quantitative PCR (RT-qPCR) was prepared using Trizol (Invitrogen, Carlsbad, CA) from 30–40 embryos; 2 μg of RNA was then used for reverse transcription with the random hexamer primers. PCR was performed in triplicates using iQ SYBR Green Super Mix. Primers are shown in supplementary material Table S1. Levels of mRNA were normalized to β-actin and calculated by using the Ct method.

### Morpholinos

At the one-cell stage, 3 ng of the following morpholinos were injected – Rps19, a specific morpholino targeting the exon 3 splicing site 5′-GCTT-CCCCGACCTTTCAAAAGACAA-3′; and 5-base-mismatch: 5′-GATTCC-TCGAACTCTCAATAGACAA-3′ (Gene Tools, Philomath, OR).

### Flow cytometry of human fetal liver cells

CD34^+^ cells were stained with Aqua-Amine (Life Technologies, Carlsbad, CA), fixed in 1.6% paraformaldehyde and permeabilized with 100% methanol. Staining was performed with antibodies against phosphorylated p53 (at residue S37) conjugated to Alexa Fluor 647 (BD Biosciences, San Jose, CA), p53 conjugated to Alexa Fluor 647, phosphorylated p53 (at residue S15) conjugated to Alexa Fluor 647, or unconjugated antibodies to phosphorylated ATM (at residue S1981), phosphorylated 53BP1 (at residue S1778), phosphorylated Chk1 (at residue S345) and phosphorylated Chk2 (at residue T68) (Cell Signaling Technology, Beverly, MA). The secondary antibodies used were conjugated to phycoerythrin (PE) and were against mouse IgGs or rabbit IgGs (Life Technologies, Carlsbad, CA). An LSR II flow cytometer (BD Biosystems, San Jose, CA) was used.

### dNTP measurement

Fifty embryos were homogenized in 1 ml of ice-cold 60% methanol, maintained overnight at −20^°^C, boiled for 3 minutes, centrifuged for 15 minutes at 17,000 ***g***, evaporated in a SVC100H SpeedVac Concentrator (Savant, Waltham, MA), resuspended in 100 μl of water and centrifuged for 15 minutes at 17,000 ***g***, 5 μl of the supernatant was then used with Klenow Fragment DNA polymerase and [^3^H]dNTP, as described previously (Mathews and Wheeler, 2009).

### ATP measurement

Twenty embryos were homogenized in 150 μl of 0.5% trichloroacetic acid, neutralized by 1 M Tris-acetate buffer, pH 7.75 and then diluted to 1.5 ml. Of this solution, 50 μl was mixed with 50 μl of rL/L reagent from ENLITEN ATP bioluminescence assay system (Promega, Madison, WI), and light output was measured by using a luminometer.

### Western blot

At 22–24 hpf, 30–40 embryos per group were lysed, and 30 μg of protein was separated on a 12% SDS-PAGE gel, transferred onto nitrocellulose membrane and probed with rabbit antibodies against phosphorylated H2A.X (at residue Ser139), phosphorylated Chk1 (at residue Ser345), phosphorylated AMPK (at residue Thr172) (Cell Signaling, Beverly, MA), or p53 (AnaSpec, Fremont, CA) followed by a horseradish peroxidase (HRP)-conjugated antibody against rabbit IgGs (Santa Cruz Biotechnology, CA). The membrane was stripped and re-probed with a mouse antibody against α-tubulin (Sigma) followed by a HRP-conjugated antibody against mouse IgGs (Santa Cruz Biotechnology). SuperSignal West Pico Chemiluminescent Substrate (Thermo Scientific, Rockford, IL) was used for detection.

### Deoxycytidine incorporation into DNA

At 10 hpf, 10 μl of 2′-Deoxycytidine[5-3H(N)], containing 16.3 Ci/mmol (Movarek Biochemicals, Brea, CA), was added to 100 embryos in 25 ml of water. At 48 hpf, genomic DNA was prepared from 25 embryos using DNA purification kit (Zymo Research, Irvine, CA). DNA was eluted in a 50 μl volume, and 40 μl of this solution was added to a liquid scintillator and the radioactivity bound to DNA was measured.

### Treatments

Deoxyadenosine, deoxyguanosine, deoxycytidine and thymidine were prepared in water at a concentration of 2.5 mM and then added to fish water to final concentrations of 10–100 μM. The ATM inhibitor KU60019, the ATM and ATR inhibitor CGK733 and the Chk1 inhibitor PF477736 were dissolved in dimethylsulfoxide to 30 mM and added to fish water at final concentrations of 10 nM, 100 nM and 20 nM, respectively.

### Statistics

Each experiment was repeated at least twice. In most cases, the results of a representative experiment are shown. Data are presented as the means of at least three measurements±s.d.; Student’s *t*-test was used for comparisons of a variable between two groups.

## Supplementary Material

Supplementary Material
