# Supplementary material for: The role of the DNA damage response in zebrafish and cellular models of Diamond Blackfan anemia
Source: Dis Model Mech. 2014 May 8;7(7):895–905. doi: 10.1242/dmm.015495 (PMC4073278; doi:10.1242/dmm.015495)
Supplement: Supplementary Material [file supp_7_7_895__index.html]

The role of the DNA damage response in zebrafish and cellular models of Diamond Blackfan anemia — Supplementary Material 

# The role of the DNA damage response in zebrafish and cellular models of Diamond Blackfan anemia

## DMM015495 Supplementary Material

**Files in this Data Supplement:**

- **Supplementary Material**
