# Supplementary material for: The role of the DNA damage response in zebrafish and cellular models of Diamond Blackfan anemia
Source: Dis Model Mech. 2014 May 8;7(7):895–905. doi: 10.1242/dmm.015495 (PMC4073278; doi:10.1242/dmm.015495)
Supplement: Supplementary Material [file supp_7.7.895_DMM015495.pdf]

## Supporting information

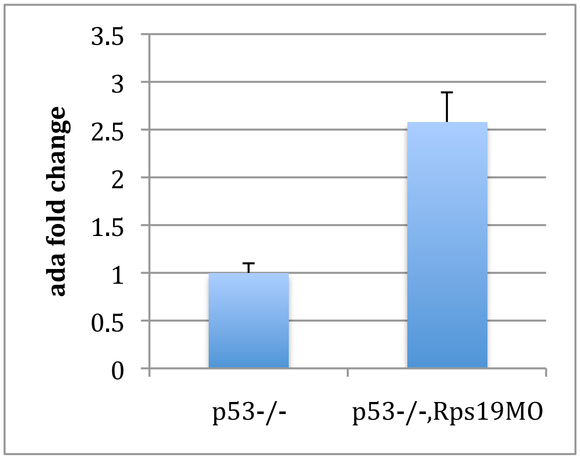


**Figure S1. Zebrafish p53-/- mutant fish injected with Rps19-specific morpholino upregulate *ada* expression.** P53-/- mutant embryos were injected with 3 ng of Rps19 morpholino at one cell stage and collected at 24 hours post fertilization (hpf). qRT-PCR. Fold change of *ada* expression in Rps19 morphants was calculated relative to non-injected p53-/- mutants.

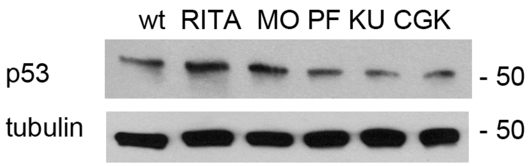


**Figure S2. Zebrafish embryos treated with p53 activating agent RITA or injected with Rps19 morpholino showed increase in p53 levels, which was ameliorated by inhibitors of ATR/ATM/Chk1/2 pathway.** As a control, wild type embryos were treated with 100μM RITA (Tocris Biosciences, Bristol, UK). Embryos were injected with 3ng of Rps19 morpholino at one cell stage; inhibitors were added at 6 hpf. 30 embryos/group were used. At 24 hpf embryos were lysed and 30 μl of proteins were used in Western blotting with rabbit antibodies to zebrafish p53 (AnaSpec, Fremont, MA) followed by HRP-conjugated anti-rabbit antibody (Santa Cruz Biotechnology, CA). The membranes were stripped and re-probed with mouse anti-alpha-tubulin antibody (Sigma) followed by anti-mouseIg-HRP antibody (Santa Cruz Biotechnology, CA).

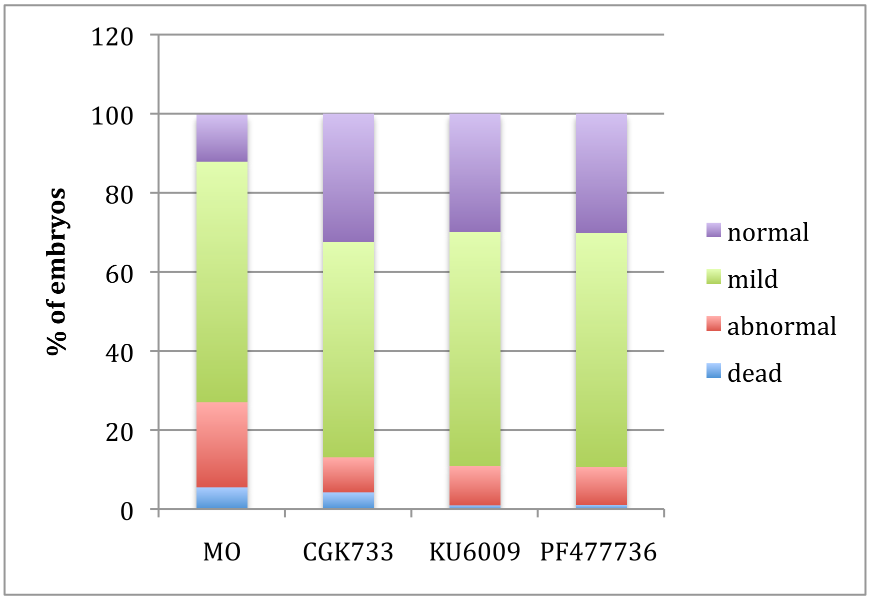


**Figure S3. Zebrafish embryos injected with Rps19 morpholino and treated with inhibitors of ATR/ATM/Chk1/2 pathway showed fewer morphological defects.** Embryos were injected with 3 ng of morpholino at one cell stage, inhibitors were added at 6 hpf. At 24 hpf embryos were scored for morphology. 60 embryos per group and 3 groups for each treatment have been used. Embryos scored as 'abnormal' had strong morphological defects such as curved body, kinks, absence of one or both eyes, very small size, or developmental arrest at 10-18 hpf. In treated groups, there was a decrease in the number of such embryos and an increase in the number of normal looking embryos. Survival also improved in groups treated by inhibitors. The table shows averages of 3 groups, which were used to make a chart, and sds.

| | dead | sd | abnormal | sd | mild | sd | normal | sd |
|---|---|---|---|---|---|---|---|---|
| MO | 5.6 | 1.3 | 21.5 | 0.45 | 60.9 | 1.15 | 11.7 | 0.93 |
| CGK733 | 4.38 | 0.85 | 8.83 | 1.18 | 54.4 | 3.61 | 32.4 | 2.5 |
| KU6009 | 1.1 | 1 | 9.97 | 1.26 | 59.1 | 0.9 | 29.8 | 1.55 |
| PF477736 | 1.2 | 1.2 | 9.56 | 1.17 | 59.1 | 2.48 | 30.1 | 2.08 |

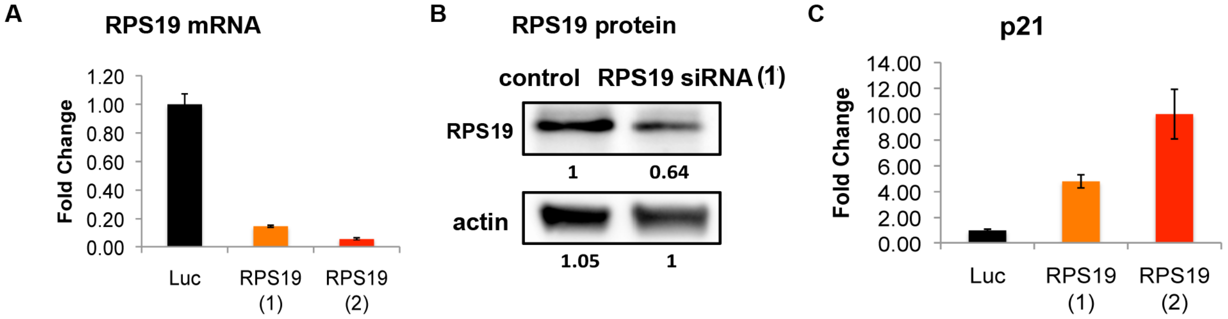


**Figure S4. CD34⁺ human fetal liver cells transduced with shRNA against RPS19 show reduced RPS19 mRNA and protein levels, and upregulation of p21**. (**A**) At day 5 after transduction, *RPS19* mRNA was reduced by ~80-90%. RT-qPCR. Luciferase shRNA was used as a control. (**B**) RPS19 protein that is incorporated into ribosomes is relatively stable; therefore, reduction of RPS19 protein level following shRNA knockdown is slower than that of mRNA. Representative Western blot of RPS19 protein in control and RPS19 shRNA treated cells. Antibodies against RPS19 (AB40833; Abcam) were used at a 1:200 dilution; beta-actin mouse monoclonal IgG2a (A5316; Sigma-Aldrich) was used as control at a 1:5,000 dilution. Densitometry was performed using Image J 1.44v software (http://rsb.info.nih.gov/ij/) to quantify the data. (**C**) p21 was upregulated in RPS19-deficient cells, day 5 after transfection, RT-qPCR.

Primers used in qPCR:
b-Actin:
Forward: CCATTGGCAATGAGCGGTT
Reverse: GCGCTCAGGAGGAGCAA
RPS19:
Forward: GCCTGGAGTTACTGTAAAAGACG
Reverse: CCCATAGATCTTGGTCATGGAGC
p21:
Forward: ATCCCGTGTTCTCCTTT
Reverse: GCTGGCATGAAGCC

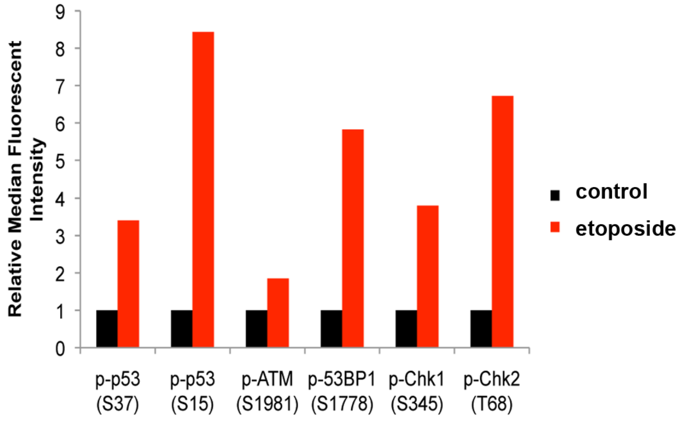


**Figure S5. Etoposide treatment induces phosphorylation of DNA damage pathway proteins in CD34⁺ human fetal liver cells.** CD34⁺ cells were transduced with a control vector containing Luciferase shRNA and cultured for 72 hours. Cells were then treated with 50uM etoposide for 2 hours to activate DNA damage response. Increased phosphorylation of p-p53 (S37), p-p53 (S15), p-ATM (S1981), p-53BP1 (S1778), p-Chk1 (S345), and p-Chk2 (T68) was observed after etoposide treatment, as measured by phospho-flow cytometry.

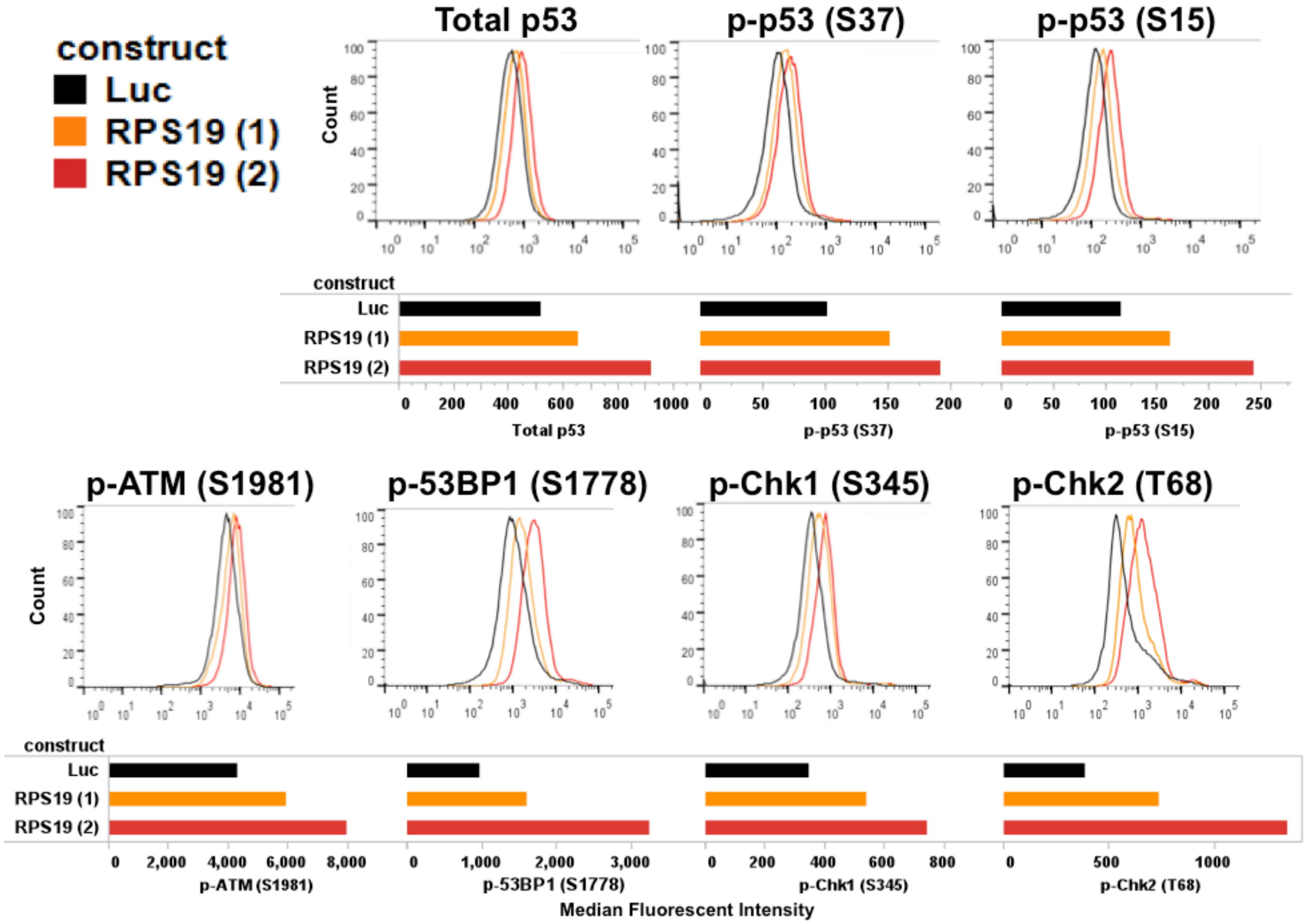


**Figure S6. Activation of DNA damage response markers induced in RPS19-knockdown cells was proportional to the degree of RPS19 downregulation.** The degree of RPS19 downregulation is shown in Fig. S4. Intracellular phosphoflow cytometry of RPS19-deficient human $CD34^+$ cells from fetal liver 5 days after transduction with lentiviral vectors expressing short hairpin RNA (shRNA) against *RPS19.* Anti-luciferase vector was used as a control. All vectors also expressed green fluorescent protein (GFP). Histograms show GFP+ gated cells from: “Luc” = control (Black); RPS19 knock down (red and orange). Bar graphs show the median fluorescent intensity obtained from the histograms for each antibody: fluorochrome conjugate. Primary antibodies to p-p53(S37), total p53, and p-p53(S15) labeled with AlexaFluor 647 have been used. The following unconjugated anti-human antibodies were used: mouse p-ATM(S1981), rabbit p53-BP1(S1778), rabbit p-Chk1(S345), and rabbit p-Chk2(T68) with a secondary labeling step with either anti-mouse Ig PE or anti-rabbit Ig PE.

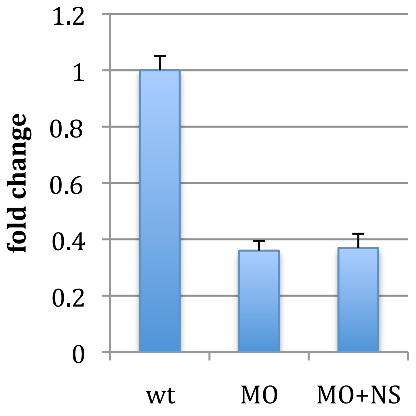


**Figure S7. The level of Rps19 downregulation by morpholino was not influenced by nucleoside treatment.** 3ng of Rps19-specific morpholino was injected at one cell stage. Half of the injected embryos were treated with 50 uM mixture of deoxyadenosine, deoxyguanosine, deoxycytidine, and thymidine starting at 3 hpf. At 24 hpf, total RNA was prepared from a pool of 30 embryos and RT-qPCR was performed with primers corresponding to exons 1 and 4 of *rps19* (forward 5'-CCAGCAATTCTGTCCAGGTCT, reverse 5'-CAGGTGGTGTAACAGTGAAA). The amount of *rps19* mRNA message was specifically reduced in embryos injected with the morpholino and was not influenced by nucleoside treatment.

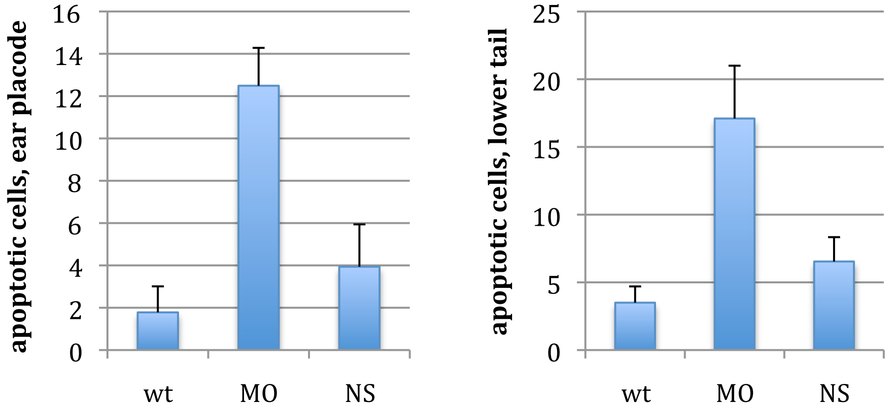


**Figure S8. Nucleoside treatment decreased apoptosis in zebrafish embryos injected with Rps19 specific morpholino.** Embryos, 80 per group, 3 groups, were injected with morpholino at one cell stage and half of them were treated with nucleosides at 3 hpf. At 30 hpf, they were stained with Acridine orange and the number of apoptotic cells in otic vesicle and in the lower part of tail were counted in wild type (wt), morphants (MO), and morphants treated with nucleosides (NS).

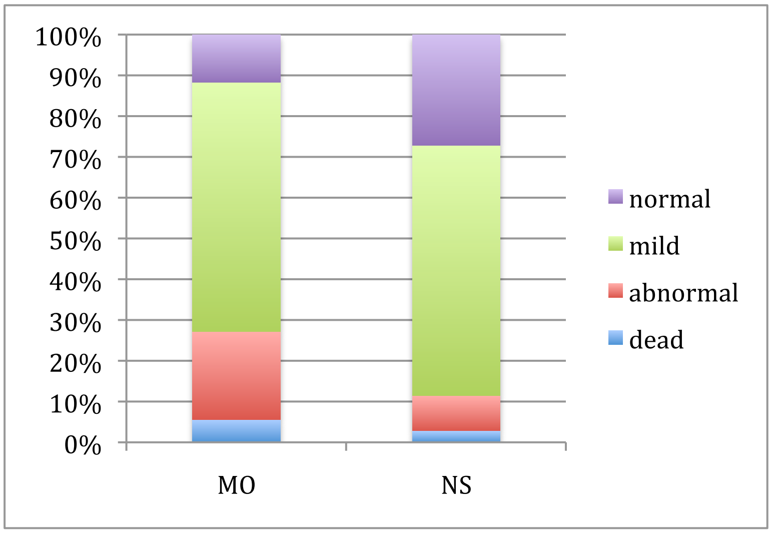


**Figure S9. Nucleoside treatment improved survival and decreased morphological defects in Rps19-deficient zebrafish embryos.** Embryos were injected with 3 ng of morpholino at one cell stage; nucleosides were added at 3 hpf. At 24 hpf embryos were scored for morphology. 60 embryos per group and 3 groups for control and treatment have been used. Scoring criteria are the same as in S2. Table shows averages for each category that were used to make a chart, and sds.

| | dead | sd | abnormal | sd | mild | sd | normal | sd |
|---|---|---|---|---|---|---|---|---|
| MO | 5.6 | 1.3 | 21.5 | 0.45 | 60.9 | 1.15 | 11.7 | 0.93 |
| NS | 2.87 | 1.1 | 8.6 | 1.9 | 61.2 | 1.71 | 27.1 | 0.85 |

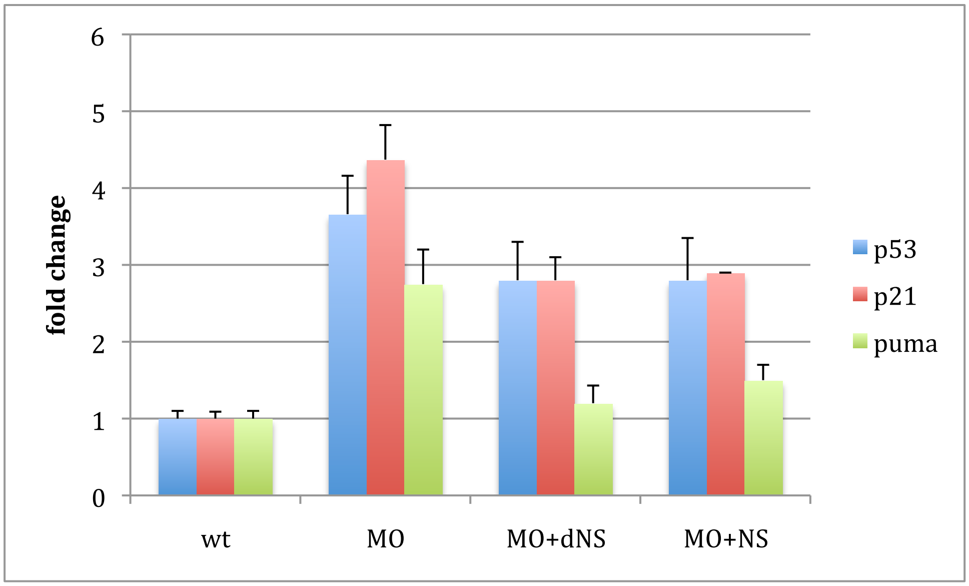


**Figure S10. Both ribonucleosides and deoxyribonucleosides decreased expression of p53 and p53 targets.** Embryos were injected with Rps19 morpholino at one cell stage, treated with nucleosides at 3 hpf, and RNA was prepared at 24 hpf from pools of 30 embryos. NS is a 50 μM mixture of adenosine, guanosine, cytidine and uridine; dNS is a 50 μM mixture of deoxyadenosine, deoxyguanosine, deoxycytidine, and thymidine. Fold change was calculated relative to expression in wild type embryos.

**Table S1**. **Primers specific to zebrafish cDNAs used in qPCR.**

| Gene | Name | Sequence |
|---|---|---|
| Actin beta | ac<br>acr | 5’-TCTCTTCCAGCCTTCCTTCCT<br>5’-CTCATCGTACTCCTGCTTGCT |
| p53 | 53f<br>53r | 5’-CTGAAGTGGTCCGCAGATG<br>5’-CGTTTGGTCCCAGTGGTGG |
| puma | zpm<br>zpmr | 5’-CCTCACATGATGCCTTCAGC<br>5’-CATTGATGGTGTCCGAGACC |
| p21 | z21<br>z21r | 5′-TGAGAACTTACTGGCAGCTTCA<br>5′-AGCTGCATTCGTCTCGTAGC |
| ada, adenosine deaminase | adf<br>adr | 5’-CCATCAAGAGAATAGCGTATG<br>5’-GCTTTCTTGTGTCCTGGGTA |
| xdh, xanthine dehydrogenase | xdh<br>xdhr | 5’-AGTTATGGTGTGGCCGTCTC<br>5’-TATACATGCCAGGTCCACGA |
| *hprt1,* hypoxanthine phosphoribosyltransferase | hprt<br>hprtr | 5’-GTGAAGAGGACACCGAGGAG<br>5’-TCATGGCTTCTCATGCTTTG |
| *tk1,* thymidine kinase | tk1<br>tk1r | 5’-TGCCTAATTCTCCACGGAAG<br>5’-ACAGGAGTAGCGCGTGTCTT |
| *dck,* deoxycytidine kinase | dck<br>dckr | 5’-AAGCCAGAGAGGTGTTTGGA<br>5’-AACGGGCACATCATTCAGAT |
| *rrm1*, ribonucleotide reductase, sub 1 | rrm1<br>rrm1r | 5’-CACATCGCTGAGCCAAACTA<br>5’-TCACTGCTGGTCGTTTTCTG |
| ppat, phosphoribosyl pyrophosphate amidotransferase | ppat<br>ppatr | 5’- GCGCAACAGAGGTCCATATT<br>5’- TGGAGCTGATCCTCTCGTCT |
| cad, carbamoyl-phosphate synthetase 2, aspartate transcarbamylase, and dihydroorotase | cad<br>cadr | 5’-TTCGTCTCATGGTGCAGAAG<br>5’-TGCGTTGAAGATGTGGACTC |
